# Antiproliferative and apoptotic effects of black turtle bean extracts on human breast cancer cell line through extrinsic and intrinsic pathway

**DOI:** 10.1186/s13065-017-0281-5

**Published:** 2017-06-20

**Authors:** Suresh Kumar, Vinay Kumar Sharma, Savita Yadav, Sharmistha Dey

**Affiliations:** Department of Biophysics, All India Institute of Medical Sciences, Ansari Nagar, New Delhi, 110 029 India

**Keywords:** Black turtle beans (*Phaseolus vulgaris*) extract, Apoptosis, Caspase 3/7, AnnexinV-FITC, Cell cycle arrest, Mitochondrial membrane potential, Bcl-2 family proteins

## Abstract

The black turtle bean (BTB) is most widely consumed legume all over the world having anticancer activity. The aim of the study was to analyse the apoptotic effects of BTB extracts on human breast cancer cell lines. Plant extract was prepared by homogenization and centrifugation. The cytotoxic effects of BTB was evaluated by MTT assay and their apoptotic effects were characterized by DNA fragmentation, nuclear staining assay, mitochondrial membrane potential analysis, annexin-V FITC and caspase 3/7 activity assay. The changes in cell cycle and gene expression of cell lines were analysed by flow cytometry and qRT-PCR, respectively. BTB extract showed cytotoxicity with IC_50_ values of 50 μg/ml in MCF-7 and MDA-MB231 cells. The caspase 3/7 was activated in the cancer cells treated with BTB extract leading to cell death by apoptosis. Moreover, there was significant increase in the expression of Bax as well as decrease in the Bcl-2 and Bcl-xL expression with in a dose dependent manner in both cells. It induces cell cycle arrest in S and G2/M phase in MCF-7 and MDA-MB231 cells, respectively. The mitochondrial membrane potential was decreased in BTB treated cells thereby transducing the apoptotic signal through the mitochondrial pathway and it also causes DNA fragmentation. Thus, it can be concluded that BTB induces the apoptosis in MCF-7 and MDA-MB-231 cells through intrinsic and extrinsic pathway and can be explored further for promising candidate to combat breast cancer. BTB extract exhibit anti-cancer activity by inducing apoptosis in breast cancer cell lines.

**Electronic supplementary material:**

The online version of this article (doi:10.1186/s13065-017-0281-5) contains supplementary material, which is available to authorized users.

## Background

The importances of plants in primary health care have been increasingly appreciated due to the growing utilization of ‘alternative’ medicines. The World health organization is promoting the use of medicinal plants, given their safety, efficacy and affordability. Most plants contain natural protectants, including flavonoids, which are powerful anti-oxidants that can also chelate metals, thereby affording protection against an array of diseases and disorders. The use of medicinal plant extracts for the treatment of human diseases is an ancient practice, which has greatly increased in recent years [1]. Natural compounds have provided many of the effective anticancer agents in current use. Over 50% of drugs used in clinical trials for anticancer activity have been isolated from natural sources or are closely related to them [2].

Black turtle beans (BTB) are a variety of common beans, belonging to the *Phaseolus vulgaris* L species of the Fabaceae family, and contain a high concentration of flavonoids. Researchers found that the darker the coat of this bean’s seeds, the higher the flavonoid contents. Such phenolic compounds, widely present in plants, inhibit or attenuate the initiation, progression and spread of cancer [3]. The high antioxidant capacity of colored beans (black, navy, pinto, red kidney and small red) has been investigated by using the oxygen radical absorbance capacity (ORAC) assay with fluorescein [4]. Black beans can enhance the body’s immune system to recognize and destroy cancer cells, as well as inhibiting the development of new blood vessels, with such angiogenesis being necessary for tumor development. Black beans also weaken the adhesiveness and invasiveness of cancer cells, thereby reducing their metastatic potentials [3].

Chronic excessive oxidative stress and inflammation are major risk factor for the development of cancer. By increasing the supply of anti-oxidant and anti-inflammatory nutrients, black beans can reduce the risk of a number of cancers, including breast and colon cancers [5]. The aim of the present study was to investigate the anticancer activity of black turtle bean extracts on the breast cancer cell lines, MCF-7 and MDA-MB231.

## Results

### Effect of BTB extract on cell viability

**Fig. 1:**
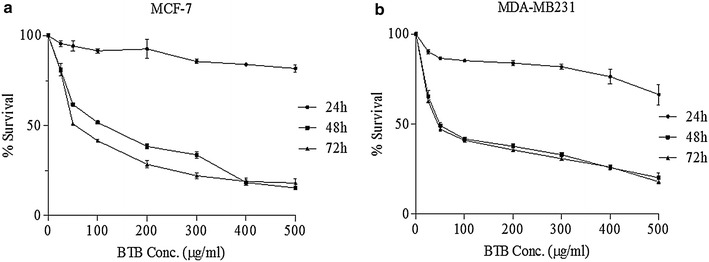
Dose-response curve showing % viability of MCF-7 (**a**) and MDA-MB231(**b**) cells at 0–500 µg/ml concentrations of BTB extract for three different time points (24, 48 and 72 h). IC_50_ was found to be 50 μg/ml in MDA-MB231 after 48 h while in the case of MCF-7 cells it was 50 μg/ml after 72 h

To explore the effects of BTB extract on MCF-7 and MDA-MB231 cells, the viability of cells was analysed by a MTT assay. After 24–72 h exposure, all treated groups showed a significant decrease in cell viability. The IC50 of the BTB extract was 50 μg/ml in MDA-MB231 after 48 h, and 50 μg/ml in MCF-7 cells after 72 h treatment (Fig. 1). The MTT assay showed that BTB extract inhibits the viability of MCF-7 and MDA-MB231 cells in a dose (50–500 μg/ml) and time dependent manner (Fig. 1a, b).

### Phase contrast microscopy for morphological analysis

**Fig. 2:**
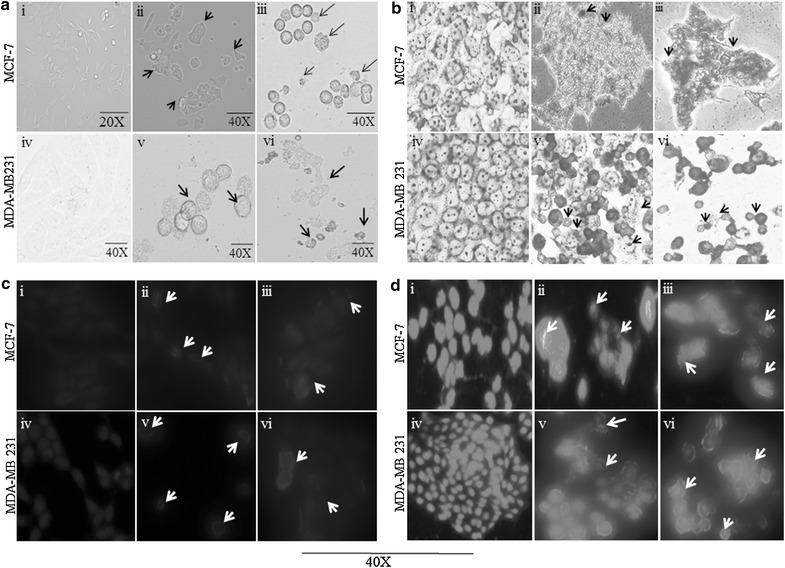
Morphological study of apoptosis in MCF-7 and MDA-MB231 induced by BTB: **a** by phase contrast microscopy (optical): (*i*, *ii*) untreated cells, (*iii*, *iv*) treated with 50 μg/ml and (*v*, *vi*) 100 μg/ml for 48 h. **b** Stained with Giemsa: (*i*, *ii*) untreated cells, (*iii*, *iv*) treated cells with 50 µg/ml, (*v*, *vi*) treated with 100 µg/ml for 48 h. **c** Stained with Hoechst 33342: (*i*, *ii*) treated with 50 µg/ml and (*iii*, *iv*) 100 µg/ml for 48 h. **d** Stained with propidium iodide: (*i*, *ii*) untreated cells, (*iii*, *iv*) treated with 50 µg/ml and (*v*, *vi*) 100 µg/ml for 48 h as shown by *arrows*

The inhibitory effect of the BTB extract was also assessed by observing morphological changes in MCF-7 and MDA-MB231 cells, using phase-contrast microscopy. The results showed a significant decrease in the number of cells following the addition of BTB extract (50 and 100 µg/ml), versus untreated cells. Furthermore, BTB extract induced morphological changes in treated cell, including cell shrinkage, membrane blebbing, cell rounding and decreased volume. However, no changes were observed in the case of normal cells (Fig. 2a). Morphological visualization with Giemsa staining showed BTB to induce apoptosis in breast cancer cell lines, as indicated by characteristic features of apoptosis, such as cell shrinkage, membrane blebbing, membrane disruption, broken nuclei and apoptotic body formations, as seen in Fig. 2b.

### Cell death assay *Hoechst 33342 staining*

Hoechst 33342 staining indicated apoptotic cells to have shrunken, condensed and fragmented nuclei after exposure of BTB extract for 48 h [Fig. 2c (ii, iii, v, vi)]. This contrasts with the untreated non-apoptotic cells, which showed a low fluorescence, smooth, flattened nuclear morphology and normal nuclei, as well as uniformly dispersed chromatin [Fig. 2c (i, ii)].

### Propidium iodide (PI) staining

Treated cells exhibited typical features of apoptosis, such as nuclei condensation, fragmentation into segregated bodies, and the formation of apoptotic bodies. The apoptotic nuclei clearly showed highly condensed or fragmented chromatin with apoptotic nuclei that were uniformly fluorescent after treatment with BTB 50 µg/ml [Fig. 2d (iii, v)] and 100 µg/ml [Fig. 2d (iv, vi)]. Untreated cells were regular and intact, with nuclei exhibiting less bright red fluorescence staining [Fig. 2d (i, ii)]. Measurement of cell death using PI (Propidium iodide) demonstrated that BTB extract caused the cell death of 42.9% (50 µg/ml) and 78.9% (100 µg/ml), compared to MCF-7 control cells, and 55% (50 µg/ml) and 74.7% (100 µg/ml) compared to MDA-MB231 cells. Thus, BTB induces the cell death in breast cancer lines in a dose-dependent manner (Additional file 1: Figure S1).

### Effect of BTB extract on cell cycle

**Fig. 3:**
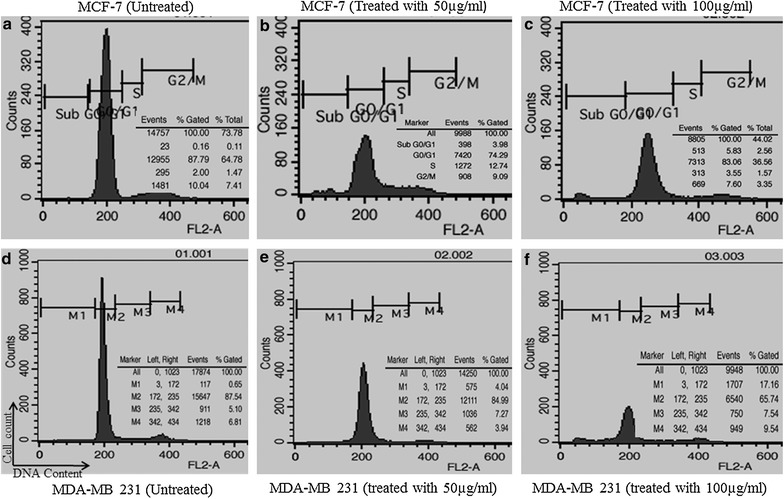
Representative histograms depicting cell cycle distribution in MDA-MB-231 and MCF-7 cultures following 48 h treatment with 50 and 100 µg/ml concentrations of BTB extract caused S and G2/M phase cell cycle arrest in MCF-7cells (**b**, **c**) and MDA-MB231 cells (**e**, **f**) with comparison to untreated control cells (**a**, **d**)

Cell cycle analysis, after 48 h treatment with BTB extract, showed a significant increase in the percentage of cells in the sub-G1 fraction, indicating apoptotic cell formation in both treated cell lines. Data also showed an enrichment of the S phase in both types of cells, as compared to untreated cells. Interestingly, MDA-MB231 cells treated with BTB extract (100 µg/ml) showed an increase in the G2/M phase, while S phase cell populations remained constant, versus untreated cells (Fig. 3). Thus, BTB extract caused S and G2/M phase cell cycle arrest in human breast cancer cells.

### BTB induced apoptosis in MCF-7 and MDA-MB-231 cells

**Fig. 4:**
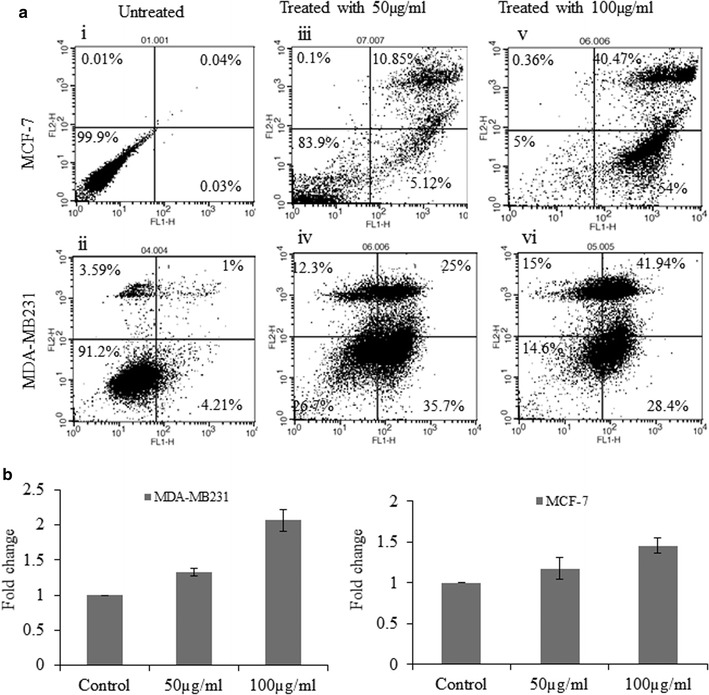
Apoptototic activity of BTB extract on human breast cancer (MDA-MB231 and MCF-7): **a** flow cytometry-based Annexin V/PI; (*i*, *ii*) untreated control, (*iii*, *iv*) treated with 50 µg/ml and (*v*, *vi*) 100 µg/ml for 48 h. **b** Caspase 3/7 activity assay: The data were expressed in fold change with respect to untreated control groups

The apoptosis marker, phosphatidylserine exposure, was examined by the Annexin V-FITC/PI assay using flow cytometry, in order to further investigate the apoptotic inducing capacity of BTB extract in breast cancer cell lines. BTB induced apoptosis in MCF-7 and MDA-MB-231 cells in a dose-dependent manner. The percentage of apoptotic cells following 50 μg/ml for 48 h was 15.97 and 60.7% MCF-7 and MDA-MB231 cells; following 100 μg/ml for 48 h, the percentage of apoptotic cells was 94.47, and 70.34%, respectively; versus 0.07 and 5.21% respectively in untreated cells. Such data indicate that cell death occurred primarily through apoptosis, following treatment with BTB extract (Fig. 4a).

### Caspase3/7 assay

To examine the molecular mechanism underlying the apoptosis process, cells were stained with aminoluciferin-labeled substrate of caspase. Cell lysates were prepared and incubated with Ac-DEVD-pNA (caspase-3/7). The reaction end products (Relative luminescence expression) were measured after 2 h incubation, as an indicant of caspase 3/7 enzyme activity. As shown in Fig. 4b, a gradual increase of caspase-3/7 activity was observed in both MCF-7 and MDA-MB-231 cells treated with 50 and 100 µg/ml of BTB extract, as compared to untreated cells (Fig. 4b). Such data indicate that BTB extract induces activation of the intrinsic caspase pathway in both breast cancer cell lines.

### Effect of BTB on mitochondrial membrane potential

**Fig. 5:**
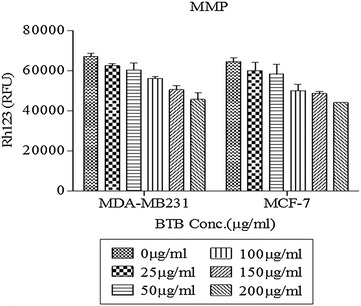
Mitochondrial membrane potential analysis by Rhodamine123 Staining: the values indicate the Rhodamine-123 fluorescence intensity in cells treated with a range of concentrations (0–200 µg/ml) for 24 h. The graph is illustrating the increased cell permeability and loss of mitochondrial membrane potential as compare to untreated control cells. The data are expressed as mean % ± SD of each group of cells from three independent experiments

To evaluate the functional status of mitochondria, the mitochondrial membrane potential was measured by staining with 10 µg/ml Rh123 dye, after treatment with BTB extract. After 24 h exposure, mitochondrial membrane potential was significantly decreased in BTB-treated MCF-7 and MDA-MB231 cells (25, 50, 100, 150, 200 µg/ml) (Fig. 5). Such data suggest that mitochondrial dependent mechanism contributed to BTB extract mediated apoptosis in breast cancer cells. BTB extract dose-dependently induced mitochondrial membrane depolarization, as characterized by decrease of mitochondrial membrane potential (Fig. 5).

### Transmission electron microscopy

**Fig. 6:**
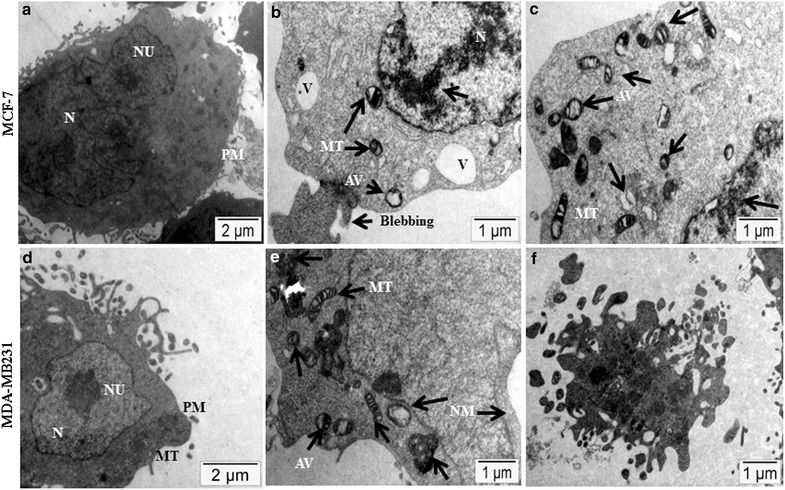
Ultrastructural features of cell death: an 48 h exposure of MCF-7 and MDA-MB231 cell lines in BTB extract in resulted in vacuole and condensed mitochondria formation, dispersed chromatin, apoptotic body formation, autophagic vesicles and membrane blebbing (**B**, **C**, **E**, **F**) with comparison to untreated control (**A**, **D**). *Arrows* indicate: *N* nucleus, *NU* nucleolus, *PM* plasma membrane, *MT* mitochondria, *V* vacuole, *AV* autophagic vesicle

Transmission electron microscopy (TEM) showed the integrity of cell membranes and many normal mitochondria in the MCF-7 and MDA-MB231 cells treated with BTB extract (Fig. 6A, D). Vacuole formation, dispersed chromatin, apoptotic body formation, autophagic vesicles, membrane blebbing, condensed mitochondria formation, and swollen mitochondria were noted in the BTB treated cells (Fig. 6B, C, E, F). Such data indicate that BTB extract resulted in ultrastructural damage, as well as inducing autophagy in MCF-7 and MDA-MB231 cells.

### Apoptosis confirmation by DNA fragmentation

**Fig. 7:**
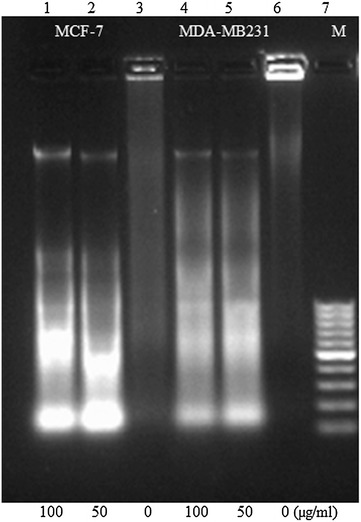
DNA fragmentation assay: *Lane 1* treated with 100 µg/ml; *2* treated with 50 µg/ml; *3* MCF-7 (untreated); *4* MDA-MB231 treated with 100 µg/ml; *5* treated with 50 µg/ml; *6* untreated; and *7* 100 bp DNA ladder

To gain further insights into the mode of cell death caused by BTB extract, its effect on the DNA fragmentation which is generally used for the detection of apoptosis, was investigated. DNA fragmentation analysis of BTB-treated cells showed a laddering pattern, which is characteristic of apoptosis, indicating internucleosomal DNA degradation (Fig. 7).

### Effect of BTB on Bcl-2, Bax and Bcl-xL expressions

**Fig. 8:**
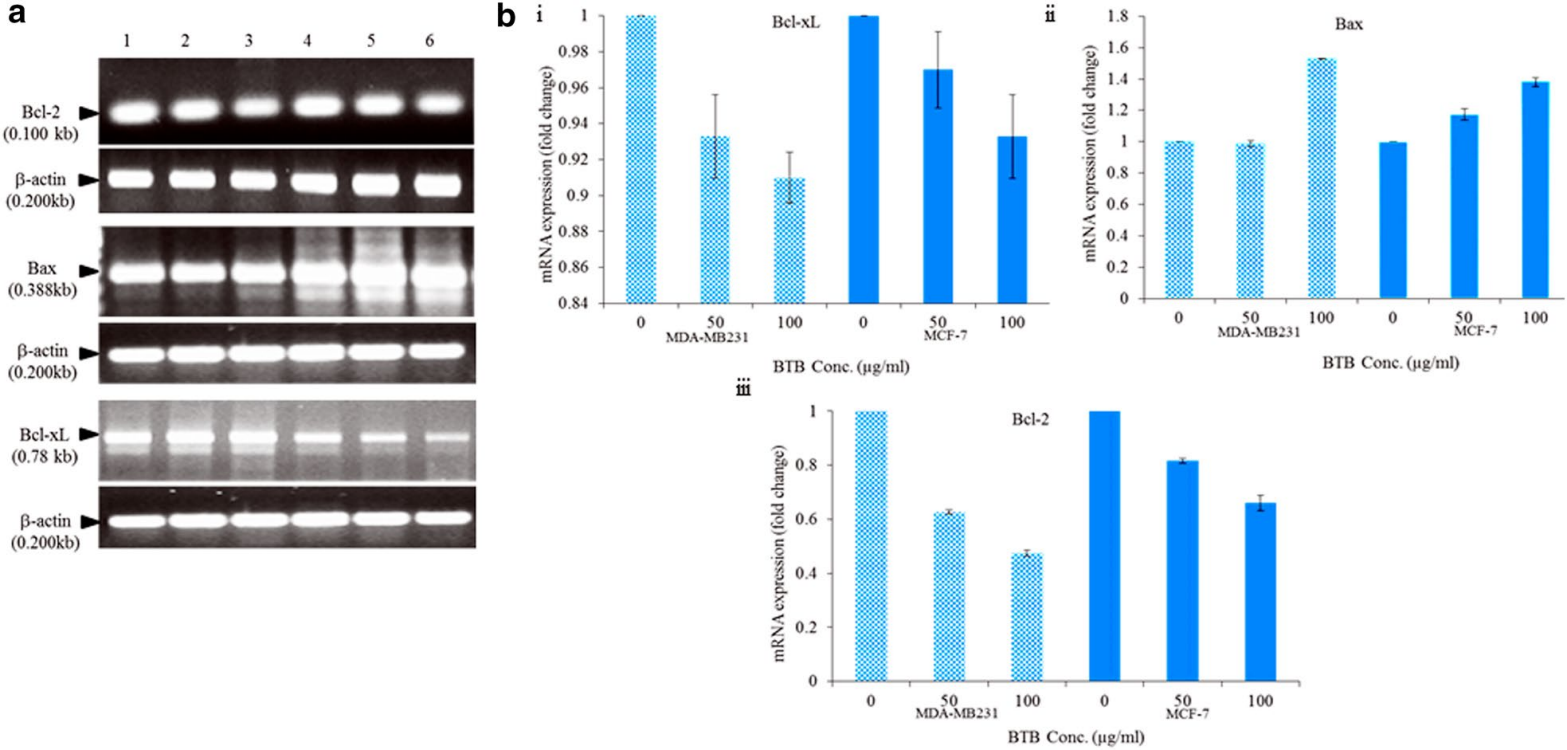
Effect of BTB extracts on Bcl-2, Bax and Bcl-xL in MCF-7 and MDA-MB231 cells (**a**) on genes (mRNA) expression by PCR in MCF-7: *lane 1* (untreated); *lane 2* and *3* treated with 50 and 100 μg/ml respectively. MDA-MB231: *lane 4* (untreated); *lane 5* and *6* treated with 50 and 100 μg/ml respectively. **b** On mRNA expression by real time PCR of intrinsic apoptotic signalling molecules on cells; change in the expression of Bcl-xL (*i*) and Bax (*ii*) genes in both breast cancer cells in a dose dependent manner, (*iii*) Bcl-2 was decreased with the increasing concentration of BTB extract

The effect of BTB extract on the expression levels of Bcl-2, Bcl-xL, and Bax genes, by RT-PCR and qRT-PCR and the clear band of amplified products, was investigated (Fig. 8a). BTB extract altered the Bcl-2, Bcl-xL, Bax gene expression after 24 h treatment in MCF-7 and MDA-MB231 cells, in a dose dependent manner [Fig. 8b (i, iii)]. Compared to untreated cells, Bax levels were markedly increased, in a dose-dependent manner [Fig. 8b (ii)]. However, the expression of Bcl-2 was decreased, as BTB extract concentration was increased [Fig. 8b (iii)]. The result suggested that BTB extract induced the change in the expression of Bcl-2 family proteins, increasing pro-apoptotic Bax and decreasing anti-apoptotic Bcl-2, thereby increasing the likelyhood of apoptosis in breast cancer cell lines.

## Discussion

Breast cancer is the most prominent cancer in women across India. According to GLOBOCAN [6], for the year 2012, an estimated 70,218 women died in India during 2012 due to breast cancer. This is higher than any other country in the world. Thus, the extracts of bioactive plants, with their known beneficial and remedial effect, is an important area of investigatation for anti-breast cancer agents. The present study explored the anticancer activity of BTB extract on human breast cancer cell lines, and the different mechanism underpinning this. To date, no study has reported the cytotoxic effect of BTB (*Phaseolus vulgaris*) extract in human breast cancer cell lines. Therefore, antiproliferative and apoptotic effects of BTB extract on MCF-7 and MDA-MB-231 cells were investigated in the present study. BTB extract induced a strong cytotoxic effect on MCF-7 and MDA-MB231 cells, in a dose dependent manner with an IC_50_ of 50 µg/ml after 48–72 h. Such data suggests that BTB extract inhibits the proliferation of different human breast cancer cells cell types, including estrogen receptor negative (MDA-MB231) and estrogen receptor positive (MCF-7) breast cancer lines. BTB extract induced morphological changes in breast cancer cell lines, including cell shrinkage, membrane blebbing, and cell rounding, *versus* untreated cells under phase-contrast microscopy and Giemsa staining. Further, BTB extract induced apoptosis in human breast cancer cell lines, as detected by Hoechst 33342 and PI nuclear staining after 48 h treatment, and also evident under fluorescent microscopy. The treated cells showed features of apoptosis such as nuclei condensation and fragmentation into segregated bodies, as well as the formation of apoptotic bodies [7].

In this study, the protein levels of caspase-3/7 were increased in breast cancer cell lines, after exposure to BTB extract. This is one mechanism by which BTB extract may contribute to the inhibition of breast carcinogenesis. Alterations in the mitochondrial membrane potential are important to the release of cytochrome c, leading to the activation of caspase cascade and subsequent cell death [8]. The loss of mitochondrial membrane potential has been considered as a critical stage in the mitochondria-mediated apoptotic pathway [9]. Our study revealed that BTB extract induced a dose-dependent depolarization of the mitochondrial membrane potential, as evidenced by the rhodamine 231 assay. Consequently, the formation of mitochondrial permeability transition pore, leading to the leakage of apoptogenic proteins, such as cytochrome c and apoptosis-inducing factor, results in caspase-dependent cell death. DNA fragmentation analysis showed that BTB extract induced DNA fragmentation in both MCF-7 and MDA-MB231 cells.

The apoptosis ratio and cell cycle arrest were analysed by flow cytometry, with different concentrations in MCF-7 and MDA-MB-231 cells. BTB extract induced cell cycle arrest in the S and G2/M phases in MCF-7 and MDA-MB231 cells. Flow cytometry analysis for apoptosis supported the cytological results, with a significant increase in Annexin-V binding following BTB extract treatment in MCF-7 and MDA-MB231 cell lines, versus untreated cells. We further provide morphological evidence in support of apoptotic pathway induction by using TEM, which morphologically discriminates the apoptotic or necrotic cell death pathways [10]. The ultrastructural analysis was also performed on MCF-7 and MDA-MB231 cells exposed to BTB extract treatments. This data again indicated the inhibitory effect of BTB extract on both cell lines with vacuole formation, condensed mitochondria formation, dispersed chromatin, apoptotic body formation, autophagic vesicles and membrane blebbing evident, as compare to untreated cells.

It is well established that Bax is positively regulated by p53 protein, and negatively controls Bcl-2 expression [11]. Apoptosis is a well-controlled process, which involves changes in the expression of an array of genes [12]. Bcl-2 family proteins have an important role in regulating cell apoptosis [13]. Over-expression of pro-apoptotic molecules, such as Bax, can accelerate cell apoptosis [14]. The inhibition of Bcl-xL and Bcl-2 expression, and their anti-apoptotic functions, can help to increase the efficiency of chemotherapeutic agents. The current investigation found that BTB extract treatment significantly increased the expression of Bax and decreased the expression of Bcl-2, and Bcl-xL, in a dose dependent manner in both MCF-7 and MDA-MB-231 cell lines. It is therefore clear that BTB extract induces apoptosis in MCF-7 and MDA-MB-231 cells, through intrinsic and extrinsic pathways. Overall, the present study shows the cytotoxic effects of BTB extract, especially on cell growth and apoptosis, in MCF-7 and MDA-MB231 breast cancer cell lines. This is the first report of BTB extract-induced breast cancer cell toxicity and programmed cell death via the apoptosis pathway in MCF-7 and MDA-MB231 breast cancer cell lines. Such data indicates that BTB extract may have therapeutic potential in the management of breast cancer. However, further investigation is required to further elucidate the molecular mechanisms underpinning BTB extract utility in the regulation of breast cancers.

## Methods

### Preparation of plant extracts from *Phaseolus vulgaris* seeds

The seeds of black turtle bean (BTB) were purchased from Gandhiana Organic Farmers’ Self Help Group (Maharashtra, India, www.gandhiana.org). Black Turtle Variety of *Phaseolus vulgaris* L. beans with Ref. No. NISCAIR/RHMD/Consult/2016/2991-18 was identified and deposited in the Raw Material Herbarium and Museum, (RHMD) NISCAIR, New Delhi, India. The seeds (60 g) were kept for swelling in 10 mM Tris–Cl buffer (pH 7.5) overnight and grinder homogenized in the same buffer. Insoluble fractions were filtered by cheese clothes and centrifuged at 13,000×*g* at 4 °C for 30 min. Before being subjected to cell culture treatments, the required concentration of crude extract named as BTB extract, was dissolved in the cell culture medium (DMEM) and filtered through 0.22 μm filter.

### Cell viability assay

To determine the effects of BTB extract on the viability of MCF-7 and MDA-MB-231 cells, an MTT assay was carried out. Cancer cells were incubated with different concentrations of extracts ranging from 25 to 500 μg/ml for 24–72 h. After the completion of the treatment time, 10 µl of 5 mg/ml MTT was added to wells and incubated for 4 h at 37 °C. Then the treatment medium was removed and 100 μl of dimethyl sulfoxide (DMSO) was added to each well to dissolve the formazan complex. The amount of colored formazan was determined by its absorbance at 570 nm in a BioTek ELISA Microplate Reader (BioTek Instrument, Inc., Winooski, VT, USA). The experiments were performed in triplicates.

### Morphological assessment of apoptotic cells

Morphological changes in MCF-7 and MDA-MB231 cells, treated with 50 and 100 µg/ml concentrations of BTB extract for 48 h, were observed under inverted contrast microscope (Nikon H600L microscope; Nikon, Japan) with suitable filter 20× and 40× magnifications consecutively. The morphological changes in the cells were also examined by staining with Giemsa (Merck, USA) for 10 min and observed under the phase contrast microscope (Nikon H600L microscope), as detailed by Chih et al. [15].

### Cell death assay

MCF-7 and MDA-MB231 cells were treated with BTB extract, at concentrations of 50 and 100 µg/ml. The cells were collected, washed by PBS and allowed to dry in situ after 48 h of treatment. Genomic DNA was stained with DNA-specific fluorescent dye; Hoechst 33342 and PI (10 µg/ml) separately for 10 min at 37 °C. The images were recorded under a fluorescence microscope (Nikon H600L microscope; Nikon, Japan [16]. Dead cells were quantified by flow cytometry (BD FACS Diva™ software).

### Cell cycle analysis

To determine the effect of BTB extract on relative cellular DNA content, cell cycle analysis was performed using propidium iodide (PI) staining via flow cytometry. Briefly, the breast cancer cells were seeded in 6-well plates at a density of 1 × 10^5^ cells per well, and then treated with 50 and 100 µg/ml of BTB extract for 48 h and stained with PI (10 µg/ml PI, 200 µg/ml RNase) for 15 min at room temperature in the dark. Untreated cells, as a negative control, were simultaneously measured. The hypodiploid DNA content of apoptotic cells were measured by quantifying the sub-G1 peak in the cell cycle pattern. The acquisition of 20,000 events per sample was recorded in a FACS Calibur (Becton–Dickinson) equipped with CELL Quest Pro software.

### Annexin V-FITC assay

BTB extract-induced apoptosis in human breast cancer cells was determined by flow cytometry using the Annexin V-FITC conjugated apoptosis detection kit (BD Biosciences, San Diego, CA). Briefly, MCF-7 and MDA-MB-231 cells (6 × 10^4^) were treated with different concentrations of BTB extract for 48 h followed by harvesting, washing with PBS, and incubation with the Annexin-V FITC (5 µl) in binding buffer at room temperature for 15 min in the dark. 5 µl of PI was added to the stained cells immediately prior to analysis, then analysed by the Cell Quest Pro software using flow cytometry (FACS Calibur, BD Bioscience).

### Caspase-3/7 activity assay

Caspase activity was determined using Caspase-Glo™ 3/7 Assay kit (Promega, Madison, WI). Cells were cultured in 96-well culture plates in 100 µl of DMEM and treated with different concentrations of BTB extract. At the end of 24 h incubation, 100 µl of assay reagent was added and incubated for 2 h at room temperature. Luminescence was measured using a Fluorescence/Multi-Detection Microplate Reader (Synergy2, BioTek Instrument, Inc., Winooski, USA).

### Measurement of mitochondrial membrane potential

The change in mitochondrial membrane potential was measured using the rhodamine 123 probe (Rh123, Sigma-Aldrich Co. MO, USA). MCF-7 and MDA-MB231 cells were seeded in 24-well culture plates at a density of 5 × 10^4^ cells/well and allowed to attach for 24 h. The media was then replaced with an equal volume of fresh DMEM containing BTB extract (25–200 µg/ml). After 48 h exposure, cultures were incubated with Rh-123 (10 µg/ml in DMSO) at 37 °C for 30 min. The change in mitochondrial membrane potential was determined using a Fluorescence/Multi-Detection Microplate Reader (Synergy 2, BioTek Instrument, Inc., VT, USA) at 485 nm excitation wavelength and 528 nm emission wavelength. Each value represents mean ± SD from triplicates.

### DNA fragmentation analysis

To confirm the apoptotic mode of cell death, DNA fragmentation assay was performed. MCF-7 and MDA-MB231 cells were treated with 50 and 100 µg/ml of BTB extract for 48 h. The lysis buffer (100 µl of 100 mM Tris pH-8.5, 5 M NaCl, 0.5 M EDTA, 0.05% TritonX-100, 10 µg/ml proteinase K and 10% SDS) was added to the pellet and incubated for 30 min on ice. The supernatant was collected in a fresh tube and mixed with 25:24:1 mixture of phenol: chloroform: isoamyl alcohol then precipitated with two equivalents of ice cold ethanol plus one-tenth equivalent of sodium acetate. This was followed by centrifugation at 12,000×*g* for 20 min. The pellet was re-suspended in 30 μl of sterile water–RNase solution (15 μg/ml RNase in sterile water) and subjected to electrophoresis in TE buffer (10 mM Tris-HCI, 1 mM EDTA, pH 8.0) on a 1% agarose gel and imaged in a Molecular Imager (Gel DocTM XR+) (BioRad, Hercules, USA).

### RNA isolation and quantitative RT-PCR

Total RNA was extracted using Ribozol reagent (AMRESCO, USA) from both untreated and treated breast cancer cells with BTB extract (50 and 100 μg/ml), after 24 h incubation according to the manufacturer’s instructions. 1 μg RNA was then reversed transcribed into cDNA in a 20 μl reaction solution containing 5X Reaction Buffer, RNase Inhibitor (20 U/µl), 10 mM deoxyribonucleotide triphosphate (dNTP) Mix, 1 μl random hexamer primer and 1 μl (200 U/µl) of MuLV reverse transcriptase (Fermentas, USA) by thermal cycler (Bio-Rad, Hercules, CA). Expression of three tumor related genes namely, Bcl-2, Bax, and Bcl-xL were studied using these cDNA as template for PCR. β-Actin was used as a control. The following oilgonucleotide primers (IDT, India) were used in the real-time qRT-PCR analysis (Table 1). Amplifications were performed in a gradient thermal cycler.

**Table 1:** List of specific primers for apoptotic genes and control

Gene	Primers	Amplification product (bps)
Bcl-2	F 5’-TGTGTGGAGAGCGTCAACC-3’ R 5’-TGGATCCAGGTGTGCAGGT-3’	100
Bax	F 5’-GATGCGTCCACCAAGAAGC-3’, R 5’AAGTCCAATGTCCAGCCCAT-3’	388
Bcl-xL	F 5’-TTGGACAATGGACTGGTTGA-3’ R 5’-GTAGAGTGGATGGTCAGTG-3’	780
β-actin	F 5’-TGGCACCCAGCACAATGAA-3’ R 5’-CTAAGTCATAGTCCGCCTAGAAGCA-3’	200

The cycling condition of the initial PCR was an activation step of 3 min at 95 °C, followed by 30 cycles of 95 °C/1 min, 72 °C/1 min and a final extension of 72 °C/10 min, for β-actin and Bcl-2 family gene. The annealing temperature of Bcl-2, Bax and Bcl-xL were 60.4, 60 and 58.4 °C, respectively. After amplification, all the PCR reactions were analysed in 1.0% agarose gel and visualized by ethidium bromide staining under UV irradiation. Gene Rular 100 bp DNA ladder (Fermentas, USA) was used as a DNA marker. Bcl-2, Bax, Bcl-xL, and β-actin mRNA expression were measured by quantitative real-time RT-PCR in an MX3005p PCR system (Stratagene, Europe). Reaction was performed using MESA Green PCR master mix containing SYBR green dye. The specificity of the amplification product was determined by melting curve analysis for each primer pairs. The data was analysed by comparative CT method and the fold change was calculated by 2^−ΔΔCT^ method described by Livak [17].

### Transmission electron microscopy

Transmission electron microscopy (TEM) was performed to identify mitochondrial morphological changes and autophagy in the MCF-7 and MDA-MB231 cell lines treated with BTB extract for 48 h. The samples were passed through propylene oxide and infiltrated in epoxy resin overnight and cured at 60 °C for 72 h. Ultracut Reichert Jung-Austria microtome was used to obtain golden color sections, thereafter stained with 2% uranyl acetate and Reynold’s lead citrate. The obtained sections were observed under a Morgagni-268 electron microscope under standard operating conditions.

### Statistical analysis

All the data are expressed as mean ± SD. The significance levels for comparison of differences were determined with a one way ANOVA, followed by Bonferroni and Dunnet post hoc tests for multiple comparisons (GraphPad Software, USA) and P < 0.05 was considered statistically significant when compared to control.

## Additional file

**Additional file 1: Figure S1.** PI staining assay showing the dead cells when treated with BTB extract (50 and 100 µg/ml) in MCF-7 and MDA-MB231 cells, respectively.
