# Supplementary figures and images for: Antiproliferative and apoptotic effects of black turtle bean extracts on human breast cancer cell line through extrinsic and intrinsic pathway
Source: Chem Cent J. 2017 Jun 20;11:56. doi: 10.1186/s13065-017-0281-5 (PMC5478552; doi:10.1186/s13065-017-0281-5)

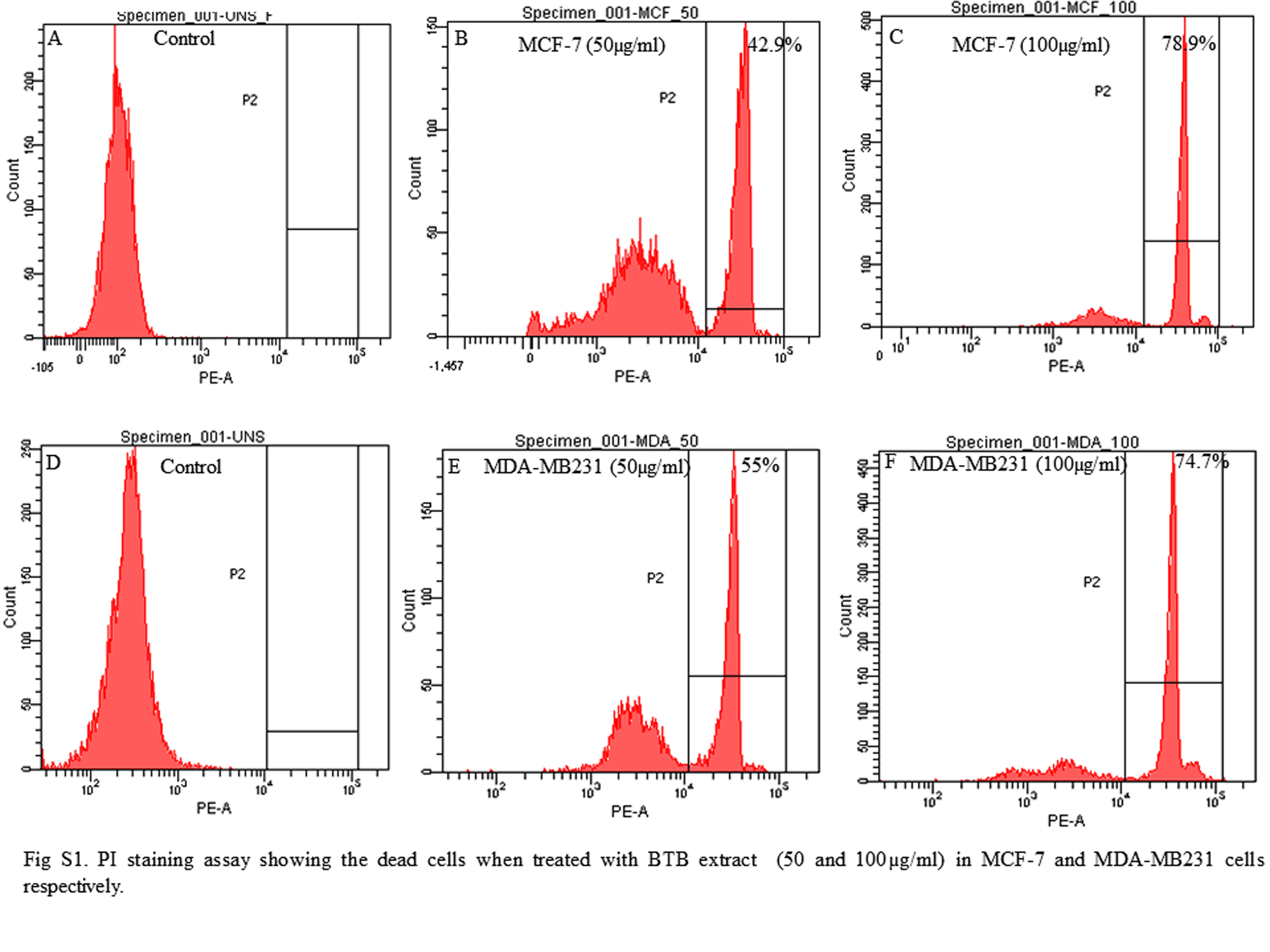

Supplement: Supplementary file 1 — Additional file 1: Figure S1. PI staining assay showing the dead cells when treated with BTB extract (50 and 100 µg/ml) in MCF-7 and MDA-MB231 cells, respectively. [file 13065_2017_281_MOESM1_ESM.tif]
